# Annual Meeting of the Illinois State Dental Society

**Published:** 1881-08

**Authors:** 


### ANNUAL MEETING OF THE ILLINOIS STATE DENTAL SOCIETY.

The seventeenth annual meeting of the Illinois State Dental Society, commenced in the city of Rock Island, May 10th, 1881.

The Society was called to order by the president, Dr. J. F. Marriner, of Ottawa. Rev. J. S. McCord, of the Methodist Church, led in prayer. The role of membership being called, the following answered to their names, viz:

| | | | |
|---|---|---|---|
| R. H. Antes | Geneseo | J. N. McCoy | Pana |
| G. V. Black | Jacksonville | W. T. Magill | Rock Island |
| T. W. Brophy | Chicago | W. H. Mills | Freeport |
| G. H. Cushing | Chicago | J. F. Marriner | Ottawa |
| E. B. Call | Peoria | C. F. Matteson | Chicago |
| J. W. Corning | Mt. Carroll | C. P. Prayne | Chicago |

| | |
|---|---|
| M. S. Dean...........Chicago | T. W. Prechett ....Whitehall |
| T. H. Gardiner.........Chicago | W. P. Richards.........Elgin |
| T. L. Gilmer..........Quincy | W. A. Stevens.........Chicago |
| H. C. Gill...........Rockford | E. D. Swain..........Chicago |
| M. F. Hand ............Joliet | G. B. Salter..............Joliet |
| J. M. Hurtt..............Peoria | E. S. Talbot........Chicago |
| A. W. Harlan.........Chicago | H. H. Townsend... ...Pontiac |
| C. A. Kitchen........Rockford | J. E. Low.............Chicago |
| R. N. Lawrence .......Lincoln | W. DeCrow ....... ...Quincy |

The following were the officers and committees during the meeting:

President.—J. F. MARRINER, Ottawa.
Vice-President.—J. A. W. DAVIS, Galesburg.
Secretary.—EDMUND NOYES, Chicago.
Treasurer.—E C. STONE, Galesburg.
Librarian.—G. H. HERRINGTON, Delavan.

STANDING COMMITTEE.

The following are the Standing Committees that served during the meeting.

Executive Committee.—T. W. BROPHY, Chicago; W. T. MAGILL, Rock Island; J. H. HURTT, Peoria.

Board of Censors.—K. B. DAVIS, Springfield; M. S. DEAN, Chicago; G. V. BLACK, Jacksonville.

Publication Committee.—E. NOYES and E. O. SWAN, Chicago.

Committee on Legislation.—G. H. CUSHING, Chicago; G. S. MILES, Jerseyville; K. B. DAVIS, Springfield.

Committee on the Infraction of the Code of Ethics.—J. N. CROUSE, Chicago; W. W. ARMSBY, Geneva; A. W. HARLAN, Chicago.

City Attorney Parks, on behalf of the city of Rock Island, extended a warm welcome to the Society and its members. He remarked that it afforded him far greater pleasure to meet them collectively, and in the present manner, than individually under the pressure of a professional engagement.

He attributed the great advance made by the dental profession during the past few years to the influence of such meetings as this. His words were fraught with encouragement, and were received with marked attention and approbation.

Dr. Black, of Jacksonville, in behalf of the association, responded to the address of welcome, and thanked the city and its representatives for the words of welcome which had been given the association. He stated that the members had every reason to remember the city of Rock Island with pleasure, as the meeting of the society here several years ago had been productive of most agreeable recollections. Dr. Black thought that the present convention would have a like result.

On motion, the Chair appointed Dr. M. S. Dean, of Chicago, and Dr. C. F. Mattison, of Chicago, a committee to extend congratulation to the Iowa State Society, now in session in Davenport, and to request them to meet with the Society during the week.

THE REPORTS OF COMMITTEES.

The reports of the Executive Committee, the Publication Committee, Committee on Legislation, Committee on Infraction of the Code of Ethics were received and adopted. The Board of Censors asked for further time.

There was a discussion as to the changes in the phraseology of the section of the constitution defining the honorary members. It was finally decided to change the section so that the same should read that all honary members who reside out of the State of Illinois, shall cease to be such members on moving into the State and continuing in the practice of the profession. Some other changes were made in the constitution.

A. W. Harlan offered a resolution which provides for the appointment of a committee of three whose duty it shall be to collect material for a dental directory of the State.

Adjourned till 2 P. M.

### FIRST DAY.

AFTERNOON SESSION.

The Society was called to order by the President, at two o'clock.

The report of Treasurer E. C. Stone, of Galesburg, was received and accepted.

G. N. Cushing, of Chicago, and Dr. Louis Ottofy, of Lebanon, were elected members.

The President, Dr. J. F Marriner, delivered his annual address, and after some general discussion of essays, the Society adjourned to allow the members to attend the night session of the Iowa State Dental Society, at Davenport.

A very pleasant and profitable evening was spent with the Iowa State Society in Davenport, at which an interesting paper on "Alveolar Ulceration, distinguished from Alveolar Abscess," was read by Dr. L. C. Ingersoll, and discussed till the adjournment.

### SECOND DAY.

### MORNING.

The entire forenoon was devoted to clinics, which were held in Armory Hall, a large and commodious place for the work. The members of the Iowa State Society spent the morning with the Illinois.

The gentlemen to conduct the morning's clinic were Dr. C. B. Rohland, of Alton; Dr. E. C. Stone, Galesburg; E. D. Swain, Chicago; R. H. Antes, Geneseo; C. H. Herrington, Deleware, and C. P. Prayou, Chicago. Dr. Rohland demonstrated the use of the many benefits to be derived from using the electro-magnetic mallet. His practical illustrations of these points were watched with interest, So, also, were the operations of Dr. Swain, of Chicago, which consisted of showing the several methods that could be employed in adjusting artificial crowns upon natural roots.

The other operations also attracted general attention of the members present.

There was in a case, provided for the purpose, in the center of the hall, a large number of preparations, books and instruments, rare, curious and useful. Especially noticeable among them were needles and other instruments, for performing operations on hare lip, cleft palate, etc., devised and made by Dr. G. V. Black. These instruments are more perfect in their adaptation to the work for which they are designed, than any heretofore used, especially in this country. The examination of the contents of this case was sufficient reward for the expense and trouble of the visit to Rock Island.

For a subject Dr. Call had a living cat. The operation performed was the forming of sutures upon the jaw, and thus explained the use of Dr. Black's instruments for a like operation.

In the afternoon, besides the business meeting, there was an essay by Dr. E. S. Talbot, of Chicago, on "The chemistry of mercury and its constitutional effects, resulting from the use of amalgam fillings and red rubber plates." This was very interesting, and the discussion that followed brought out some new thoughts on the subject.

### FOURTH DAY.

### MORNING SESSION.

The meeting was called to order, and the minutes of the last session were read. After some routine business discussion was resumed.

On Dr. C. W. Spaulding's essay, entitled: "Operative Dentistry." This was participated in by Drs. Taylor, of Streator; French, of Pittsburg; Brophy, of Chicago; Spaulding of St. Louis; Templeton, of Pittsburgh; Black, of Jacksonville; Harlan, of Chicago; Hanford, of Rockford; Town, of Pontiac, and Davis of Springfield.

### SECOND DAY.

### AFTERNOON.

After the transaction of miscellaneous business, several essays were read, and discussions had upon them, a full report of which will be given in the transactions.

The papers read were:

Treatment of teeth containing dead and dying pulps; also, the treatment of alveolar abscess—Dr. H. H. Townsend, Pontiac.

Mechanical dentistry; its past and future—Dr. L. P. Haskell, Chicago.

The development of the enamel—Dr. M. S. Dean, Chicago.

Fractures of the inferior maxilla—Dr. T. L. Gilmer, Quincy.

Dental hygiene—Dr. W. P. Richards, Elgin.

The chemistry of mercury and its constitutional effects, resulting from the use of amalgam fillings of red rubber plates—Dr. E. S. Talbot, Chicago.

What must be the preparation for the successful practice of dentistry in the future—Dr. C. A. Kitchen, Rockford.

Operative dentistry,—Dr. G. S. Miles, Jerseyville.

Report on the saliva—Dr. C. W. Spalding, St. Louis.

Adjourned.

### THIRD DAY.

The forenoon was devoted to clinics, which were conducted by Drs. E. B. Call and E. J. Green, of Peoria; E. B. David, of Aledo; W. W. Dean, of Lacon; W. N. Morrison, of St. Louis, and D. B. Freeman, of Chicago.

Dr. Call demonstrated most successfuly his method of adjusting gold crowns upon natural roots. Dr. Green illustrated the use of the soft or non-cohesive gold, and made several experiments with that filling.

Dr. Morrison extracted a tooth from a patient, filled it and afterwards replaced or replanted it in its former location in the patient's mouth.

Following this came Dr. Spalding's essay, "Suggestions relative to the causes of rapid Dental Decay," Then Dr. A. W. Harlan read his essay on "Characteristics of saliva in scrofulous patients."

Quincy was selected for the next meeting of the Society.

Officers for the ensuing year were elected as follows:

President.—A. W. Harlan, Chicago.
Vice-President.—W. T. Magill, Rock Island.
Secretary.—E. Noyes, Chicago.
Treasurer.—E. C. Stone, Chicago.
Librarian.—H. H. Townsend, Pontiac.

### COMMITTEES.

Executive Committee.—E. S. Talbot, Chicago; S. M. Sturgiss, Quincy; J. M. Downing, Macomb.

Board of Examiners.—G. V. Black, Jacksonville; K. B. Davis, Springfield; M. S. Dean, Chicago.

Publication Committee.—E. Noyes, Chicago; E. D. Swain, Chicago.

Committee on Legislation.—T. W. Brophy, Chicago; E. D. Swain, Chicago; G. S. Miles, Jerseyville; K. B. Davis, Springfield; A. W. Harlan, Chicago,

Committee on Infraction of the Code of Ethics.—F. H. Gardner, Chicago; W. B. Woodward, Peoria; G. D. Sitherwood, Bloomington.

Resolutions of thanks were voted the Mayor, the Rodman Rifles, the press and the hotel proprietors for courtesies extended.

Dr. M. S. Dean, of Chicago, was named as a delegate to the Medical Congress at London, next August.

A vote of thanks was tendered Dr. Cushing for his labors on the legislative committee.

With the reading of the minutes of the session, the convention adjourned to meet in Quincy, May, 1882.

